# Genome-Wide Association Study (GWAS) of dental caries in diverse populations

**DOI:** 10.1186/s12903-021-01670-5

**Published:** 2021-07-26

**Authors:** Rasha N. Alotaibi, Brian J. Howe, Jonathan M. Chernus, Nandita Mukhopadhyay, Carla Sanchez, Frederic W. B. Deleyiannis, Katherine Neiswanger, Carmencita Padilla, Fernando A. Poletta, Ieda M. Orioli, Carmen J. Buxó, Jacqueline T. Hecht, George L. Wehby, Ross E. Long, Alexandre R. Vieira, Seth M. Weinberg, John R. Shaffer, Lina M. Moreno Uribe, Mary L. Marazita

**Affiliations:** 1 Department of Oral and Craniofacial Sciences, School of Dental Medicine, University of Pittsburgh, Pittsburgh, PA USA; 2 Center for Craniofacial and Dental Genetics, School of Dental Medicine, University of Pittsburgh, Pittsburgh, PA USA; 3 Dental Health Department, College of Applied Medical Sciences, King Saud University, Riyadh, Saudi Arabia; 4 Department of Family Dentistry, College of Dentistry, University of Iowa, Iowa City, IA USA; 5 The Iowa Center for Oral Health Research, College of Dentistry, University of Iowa, Iowa City, IA USA; 6 Department of Human Genetics, Graduate School of Public Health, University of Pittsburgh, Pittsburgh, PA USA; 7 UC Health Medical Group, Colorado Springs, Denver, CO USA; 8 Department of Pediatrics, College of Medicine, University of the Philippines, Manila, Philippines; 9 ECLAMC/INAGEMP At Center for Medical Education and Clinical Research (CEMIC-CONICET), Buenos Aires, Argentina; 10 Department of Genetics, Institute of Biology, Federal University of Rio de Janeiro, Rio de Janeiro, Brazil; 11 Dental and Craniofacial Genomics Core, School of Dental Medicine, University of Puerto Rico, San Juan, PR USA; 12 Department of Pediatrics, University of Texas Health Science Center At Houston, Houston, TX USA; 13 Department of Health Management and Policy, College of Public Health, University of Iowa, Iowa City, IA USA; 14 Lancaster Cleft Palate Clinic, Lancaster, PA USA; 15 Department of Orthodontics, School of Dentistry, University of Iowa, Iowa City, IA USA

**Keywords:** Genomics, Genetics, Dental caries, Ethnicity

## Abstract

**Background:**

Dental caries is one of the most common chronic diseases and is influenced by a complex interplay of genetic and environmental factors. Most previous genetic studies of caries have focused on identifying genes that contribute to dental caries in specific ethnic groups, usually of European descent.

**Methods:**

The aim of this study is to conduct a genome-wide association study (GWAS) to identify associations affecting susceptibility to caries in a large multiethnic population from Argentina, the Philippines, Guatemala, Hungary, and the USA, originally recruited for studies of orofacial clefts (POFC, N = 3686). Ages of the participants ranged from 2 to 12 years for analysis of the primary dentition, and 18–60 years for analysis of the permanent dentition. For each participant, dental caries was assessed by counts of decayed and filled teeth (*dft*/DFT) and genetic variants (single nucleotide polymorphisms, SNPs) were genotyped or imputed across the entire genome. Caries was analyzed separately for the primary and permanent dentitions, with age, gender, and presence/absence of any type of OFC treated as covariates. Efficient Mixed-Model Association eXpedited (EMMAX) was used to test genetic association, while simultaneously accounting for relatedness and stratification.

**Results:**

We identified several suggestive loci (5 × 10^−8^ < *P* < 5 × 10^−6^) within or near genes with plausible biological roles for dental caries, including a cluster of taste receptor genes (*TAS2R38, TAS2R3, TAS2R4, TASR25*) on chromosome 7 for the permanent dentition analysis, and *DLX3* and *DLX4* on chromosome 17 for the primary dentition analysis. Genome-wide significant results were seen with SNPs in the primary dentition only; however, none of the identified genes near these variants have known roles in cariogenesis.

**Conclusion:**

The results of this study warrant further investigation and may lead to a better understanding of cariogenesis in diverse populations, and help to improve dental caries prediction, prevention, and/or treatment in future.

**Supplementary Information:**

The online version contains supplementary material available at 10.1186/s12903-021-01670-5.

## Background

Dental caries is a common multifactorial disease, in which environment and genetics each play important roles. Dental caries is a global public health problem and if left untreated, can lead to serious complications including pain, infection, abscess and loss of teeth [1] There are at least 2.3 billion people globally affected with caries of the permanent dentition, and more than 530 million children with caries of the primary dentition [2]. The 2010 Global Burden of Disease, Injury and Risk Factors (GBD) study estimated that oral diseases, such as untreated dental caries and severe periodontitis, accounted for 15 million disability-adjusted life-years (DALYs) globally among people older than 60 years old; and untreated dental caries in permanent dentition was the most common condition in the entire 2010 GBD study (global prevalence for all ages is 35%) [3].

The role of environmental risk factors in causing dental caries is very well established, and includes factors such as lack of fluoride, poor oral hygiene habits, and consuming a diet high in sucrose [4, 5]. In addition, disparities in terms of income, educational level and access to dental services in areas such as Appalachia in the United States could be associated with an increased risk of oral diseases, especially dental caries [6]. Moreover, a systematic review study indicated that socioeconomic factors, such as educational level, occupation and income, are highly associated with dental caries among adults [7].

Over the last decade, human genetic studies have made significant progress identifying genetic risk factors associated with dental caries. Previous genome-wide association studies (GWAS) of dental caries, summarized in Table 1, have reported numerous associations. However, these prior studies were limited to a single ethnic group and/or in a single dentition [8–10]. Therefore, the aim of the current GWAS study is to investigate both primary and permanent dentitions in a multiethnic cohort of children and adults.

**Table 1:** Genome-wide studies of dental caries

Study	Sample size	Dental caries phenotype	Genes	Genome-wide significant associations
Shaffer et al. 2011	1305 white children, age 3–12	Binary affection status in primary dentation	*ACTN2, EDARADD, MPPED2, MTR,* and *LPO*	No
Wang et al. 2012	7443 whites Adult, age 17–89	Permanent decayed, missing, filled surfaces index (DMFS)	*RPS6KA2, ISL1, TLR2 RHOU, FZD1, PTK2B,* and *ADMTS3,*	No
Shaffer et al. 2013	920 whites Adult, ages 18–75	Permanent cluster- based partial DMFS	*LYZL2, AJAP1*	Yes
			*ABCG2, PKD2, the* dentin/bone *SCPP* sub-family*, EDNRA, TJFBR1, NKX2-3, IFT88, TWSG1, IL17D,* and *SMAD7*	No
Zeng et al. 2013	1017 whites Adult, age 14–56	Permanent decayed and filled teeth (dft) stratified to generate df-pitt and fissures (dfPF)and df-smooth surface (dfSM)	*BCOR, BCORL1, INHBA, CXCR1* and *CXCR2*	No
Zeng et al. 2014	1006 white children, age 3–14	Primary decayed and filled teeth (dft) stratified to generate df-pitt and fissures (dfPF)and df-smooth surface (dfSM)	*KPNA4, ITGAL, PLUNC* family genes	Yes
			*MPPED2, AJAP, and PRS6KA2*	No
Haworth et al. 2018	19,003 Primary analysis; 13,353 Permanent analysis European ancestry meta-analysis age 2.5–18	Presence or absence of treated or untreated caries	*ALLC, NEDD9* for primary and permanent dentition respectively	Yes
Shungin et al. 2019	GLIDE and UKB (n = 26,792)	DMFS, DFSS, Nteeth	*C5orf66, CA12*	Yes
			*KRTCAP2, WNT10A, FGF10, HLA, FOXL1, PBX3, MAMSTR*	No

## Methods

### Sample

The study sample (N = 3686) comprised individuals recruited as part of a large international study of orofacial clefting. Individuals recruited for this study included affected cases, their unaffected family members, and unrelated healthy controls from the United States (Colorado, Iowa, Pennsylvania, Texas, and Puerto Rico) and internationally (Argentina, the Philippines, and Hungary). All participant provided written informed consent for themselves and for children younger than 18 years, informed consent was obtained from their parents or their legally authorized representative. Local ethical approval was obtained at each site and all methods in this study were performed in accordance with the Institutional Review Board policies and guidelines of the University of Pittsburgh and all of the other sites.

The total sample for the primary dentition analysis included 1116 children (age 2–12 years) and 2570 adults for the permanent dentition analysis (ages 18–60 years). Table 2 summarizes the sex and cleft status breakdown for each analysis. Since our participants came from multiple sites, Table 3 summarizes the distribution of participants for the different recruitment sites.

**Table 2:** Basic characteristics of the study cohorts

Male, n (%)	Female, n (%)	Age, range Mean ± SD	No cleft	With cleft	dft/DFT, range Mean ± SD	Total
*Primary*						
619 (55.5%)	497 (44.5%)	2–12 6.9 ± 2.6	687	429	0–20 2.6 ± 3.6	1116
*Permanent*						
1001 (38.9%)	1569 (61.1%)	18–60 34.4 ± 9.9	2408	162	0–25 5 ± 4.3	2570

**Table 3:** Distribution of participants across different sites

Site	Primary	*dft* Mean ± SD	Permanent	DFT Mean ± SD
*US sites*				
Colorado	26	1.42 ± 1.55	32	8.12 ± 3.87
Iowa	225	0.65 ± 1.48	358	3.51 ± 3.56
Pittsburgh	78	1.16 ± 2.54	147	7.48 ± 5.10
Texas	130	2.55 ± 3.21	196	4.74 ± 4.69
Puerto Rico	55	2.09 ± 2.80	121	8.53 ± 4.06
Total	514 (46.1%)	1.40 + 2.47	854 (33.2%)	5.36 ± 4.66
*Non-U.S sites*				
Hungary	213	2.31 ± 2.94	565	6.45 ± 4.48
Argentina	106	2.46 ± 3.09	246	4.32 ± 3.95
Philippines	159	6.35 ± 4.86	563	4.20 ± 3.71
Guatemala	124	3.76 ± 3.34	342	3.26 ± 3.67
Total	478 (53.9%)	3.70 ± 4.00	1374 (66.8%)	4.78 ± 4.19
Total across all sites	1116	2.6 ± 3.6	2570	5 ± 4.3

### Dental caries assessment

The same data collection protocols were used for every site. Dental caries scores (*dft*/DFT) were assessed by trained dentists or dental hygienists, with data collected either through in-person dental examinations and/or intraoral photos (at least 5 per participant, maxillary and mandibular occlusal, right and left lateral, anterior biting) to appropriately cover the entire oral cavity, as previously reported [11]. The *dft*/DFT index was calculated as the total number of teeth with decayed, and/or filled/restored surfaces. In addition, questionnaires recording dental history were collected for all participants in the study. We used the *dft*/DFT index instead of *dmft*/DMFT due to incomplete information regarding missing teeth for the majority of the participants. Note that for the children, primary dentition caries scores (*dft)* were obtained only from the primary teeth, any permanent teeth in these children were not included. Table 3 summarizes the dental caries mean scores for the different recruitment sites in analyses of both the primary and permanent dentitions.

All of the training and calibration for the intra-oral photos and the in-person dental exam was completed prior to the start of data collection. Each photo rater (BJH, LMU, and ARV) rated randomly selected participants (n = 15) for calibration twice. Results from ratings by LMU and ARV were calibrated against the gold-standard rater, BJH (note BJH’s intra-rater reliability (kappa) = 0.95)). The Inter-rater reliability (kappa) between all the 3 raters = 0.91–0.93. Data from 158 participants who had both intra-oral photos and in-person dental exams were used to measure the consistency between in-person dental exams and the ratings from intra-oral photos; the results showed excellent agreement (kappa > 90%) between the two methods [11].

### Genotyping

Participants were genotyped on the Illumina Human610-Quadv1_B BeadChip (Illumina, Inc., San Diego, CA, USA) and Illumina Infinium II assay protocol by the Center for Inherited Disease Research (CIDR) at Johns Hopkins University. Data cleaning and quality assurance procedures were conducted in conjunction with the CIDR data cleaning center at the University of Washington. 529,285 SNPs (single nucleotide polymorphism) were genotyped, and additional SNPs were imputed based on the 1000 Genomes Project reference panel resulting in 35,153,445 SNPs [12]. Of those, 8,859,951 SNPs passed quality control and analysis filters (participant call rates > 90%; SNP call rates > 99%; Hardy–Weinberg p values > 0.0001; MAF > 2%) and were therefore analyzed in this study.

### Statistical analysis

#### Genetic analyses

Caries scores in the primary dentition (*dft*) were analyzed separately from caries scores in the permanent dentition (DFT), both as quantitative traits. The analysis of *dft* in the primary dentition included participants of aged 2–12 years, and the analysis of DFT in the permanent dentition included participants aged 18–60 years. The statistical software used, namely, the Efficient Mixed-Model Association eXpedited (EMMAX) [13] uses a variance component-based method for testing the SNP main effects along with a genetic relationship matrix (GRM) and covariates (in this case, sex, recruitment site, age, age^2^, and cleft status). Results of modeling of those covariates to determine if they are needed to be included to adjust our primary *dft* and permanent DFT analyses are shown in Table 4. Adjustment for those covariates were included in the final analyses. The genomic inflation factor (λ), and Manhattan plots were generated using the R v3.4.1 statistical analysis environment (R Core Team, 2019). Given the issue of multiple comparisons, conservative p-value thresholds for genome-wide statistical significance were set to *P* < 5 × 10^−8^. The p-value thresholds for suggestive significance were set equal to 5 × 10^−8^ < *P* < 1 × 10^−6^.

**Table 4:** Regression results for the covariates

Phenotype	Covariate	Beta	SE	*P*
Primary *dft*	Age	1.69641	0.19039	< 2e−16***
	Age^2^	− 0.12954	0.01357	< 2e−16***
	Sex	− 0.10053	0.19735	0.611
	Site	2.27537	0.19744	< 2e−16***
	Cleft status	0.80861	0.20120	6.24e−05***
Permanent DFT	Age	0.3832854	0.0531329	7.14e−13***
	Age^2^	− 0.0039532	0.0007238	5.17e−08***
	Sex	0.6350039	0.1723444	0.000234***
	Site	0.3680415	0.1865084	0.048566*
	Cleft status	1.4312329	0.3496957	4.39e−05***

Significant P values codes: 0 ‘***’ 0.001 ‘**’ 0.01 ‘*’ 0.05

#### Bioinformatic approaches for the genetic results

Different bioinformatics and visualization tools were used in this study to interpret the GWAS results at both the SNP and gene level. Loci/genes of interest were identified and reported based on physical proximity of ± 500 kb windows from the lead SNP at each of the loci associated with dental caries. We used LocusZoom [14], to visualize the associations results in the regions around the lead SNPs at each locus. To gain more information regarding the biological consequences of the lead SNPs in the risk loci, variant annotation tools, such as HaploReg, were used to annotate regulatory information, such as enhancer/promoter regions, expression quantitative trait loci (eQTLs), and transcription factor binding sites [15]. Other regulatory databases, such as the Genotype-Tissue Expression (GTEx) [16], and Encyclopedia of DNA Elements [17] were used, too, to help in interpreting the associations results.

## Results

### Cohort characteristics and covariate modeling

The total study sample comprised 3686 genotyped and phenotyped individuals. The primary dentition cohort (n = 1116) included 616 males (55.5%), and 497 females (44.5%) with an age range of 2–12 years and a mean age of 6.9 years (Table 2), 46.1% of the participants were from U.S recruitment sites, and 53.1% from international sites. The permanent dentition cohort (n = 2570) included 1001 males (38.9%), and 1569 females (61.1%) with an age range of 18–60 years and a mean age of 34.4 years (Table 2), with 33.2% of the participants from U.S recruitment sites, and 66.8% of participants from international sites. General linear regression analysis (details in Table 4) showed that age, recruitment site, cleft status (affected vs. unaffected with cleft) and sex had an impact on the DFT and *dft* mean scores; therefore, these factors were included as covariates in the genetic analyses.

### Genetic results

**Fig. 1:**
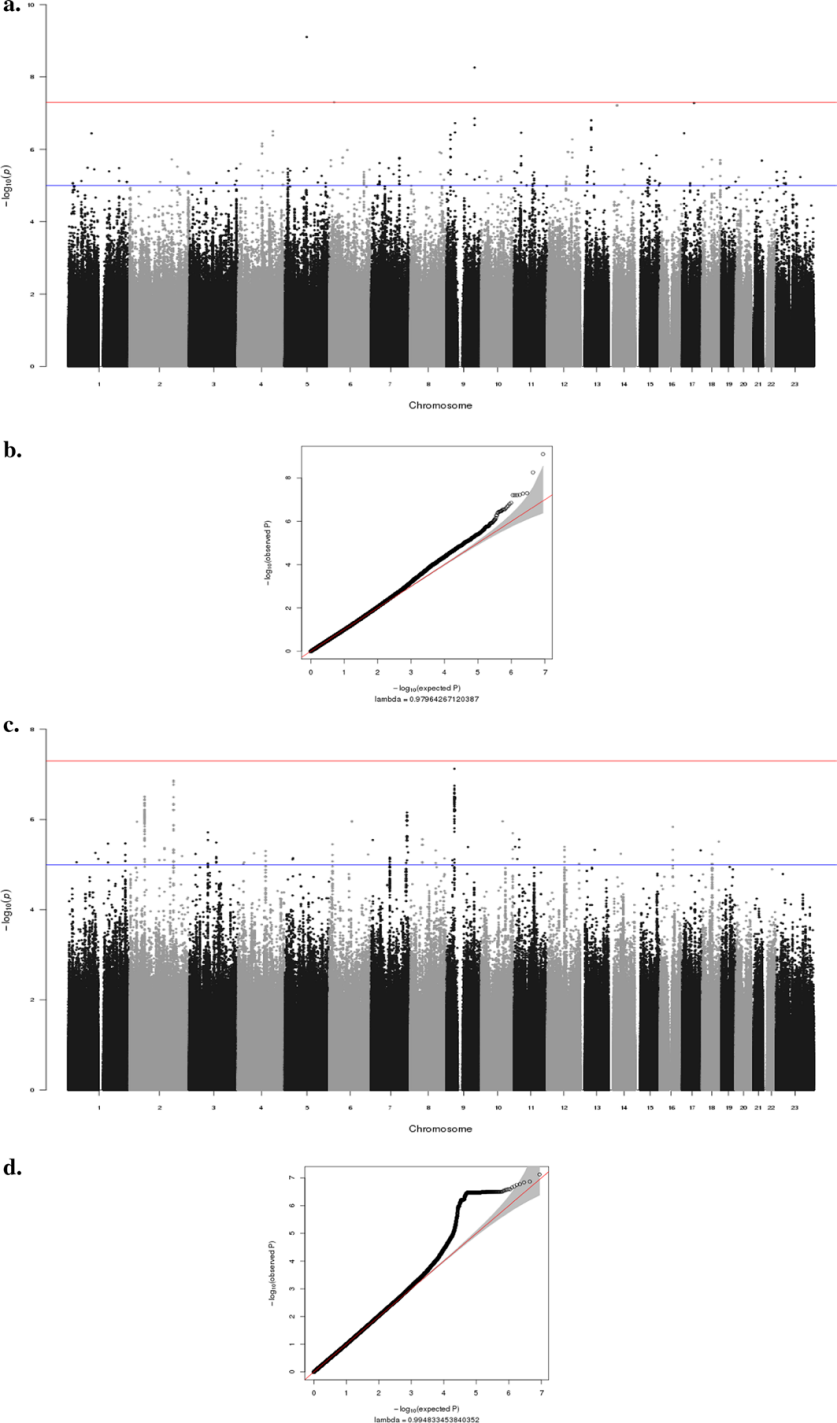
Manhattan plots and (Q-Q) plots showing GWAS results for the analyses. Red lines represent thresholds for genome-wide significance (*p* value < 5 × 10^−8^). Blue lines represent thresholds for suggestive significance (*p* value < 5 × 10^−6^). **a** Manhattan plots for primary *dft* show negative log10-transformed p values (y-axis) across the whole genome (x-axis). Genotyped and imputed SNPs are plotted together. **b** The quantile–quantile plot (Q-Q) for GWAS of Primary *dft*. The genomic inflation factor (λ) is 0.98. **c** Manhattan plots for permanent DFT show negative log10-transformed p values (y-axis) across the whole genome (x-axis). Genotyped and imputed SNPs are plotted together. **d** The quantile–quantile plot (Q–Q) for GWAS of permanent DFT. The genomic inflation factor (λ) is 0.99

Manhattan plots illustrating the GWAS results for the primary *dft* and permanent DFT are shown in Fig. 1a, c. The genomic inflation factor (λ) for the primary dentition analysis was 0.98, and for the permanent dentition analysis was 0.99, indicating no inflation of p values (Fig. 1b, d). Note that associations identified under the GWAS approach do not specify which SNP at that locus is the causal SNP (cause the association) or which gene is affected by the causal SNP. Here we report any known biological functions of genes near our significant and suggestive results that have plausible roles in dental caries or have some biological relevance to tooth development and/or oral health.

#### Primary dentition

For the primary *dft*, SNPs at multiple loci exceeded the threshold for genome-wide significance (*P* < 5 × 10^−8^) and others exhibited suggestive significance (5 × 10^−8^ < *P* < 1 × 10^−6^). The biological roles of potential candidate genes at these loci are described in Table 5. Genes at two of the genome-wide significant loci have no known role in cariogenesis and warrant further investigation. One of the lead SNPs (rs113021760, P = 3.36 × 10^−8^), is located in the intron of the *SUSD1* gene (Sushi Domain Containing 1), so it might play a regulatory role. In addition, this SNP shows enhancer chromatin marks in different tissues, including osteoblasts, which are found in the periodontal ligament and alveolar bone in the oral cavity.

**Table 5:** Significant and suggestive SNPs across the analyses

Lead SNP	Chr band	BP	Beta	*P*	Nearby Gene(s)	dbSNP Annotation	Biological Role*
*Primary analysis*							
rs80177293	5q14.3	88,865,345	− 2.91	8.23e−10	*MIR3660*	Non-coding	Essential for post- transcriptional regulation of gene expression in different organism [Hansen, et al. 2010]
rs113021760	9p23	114,805,385	− 2.67	3.36e−09	*SUSD1* *UGCG*	Intronic	A protein coding gene that has been associated with Venous thromboembolism (VTE), a cardiovascular disease [Tang et al. 2013] Involved in the biosynthesis of glycosphingolipid (Ichikawa, et al. 1996)
rs75833698	6p22.3	19,866,520	− 2.17	4.93e−08	*ID4*	Non-coding	Inhibitor of DNA binding 4 has been, regulate different cellular processes [Benezra, et al. 2001]
rs16948495	17q21.33	48,005,748	− 2.15	5.16e−08	*DLX3* *DLX4*	Non-coding	Mutations in this gene have been associated with tricho-dento-osseous syndrome (TDO), which cause enamel hypoplasia [Y.li et al. 2015] Play a role in forebrain and craniofacial development. Mutations cause non-syndromic orofacial cleft [Wu et al. 2015]
rs75459295	14q13.1	34,767,100	− 2.26	6.08e−08	*SPTSSA*	Non-coding	Involved in sphingolipid biosynthesis (Han, et al. 2009)
chr9:35,753,170	9p13.3	35,753,170	− 2.27	1.89e−07	*CA9* *TLN1*	Non-coding	Participates in a variety of biological processes, including regulation of pH, and can be found in gastrointestinal mucosa [Yang et al. 2017] Plays a role migration of various cell types, including fibroblasts and osteoclasts [Monkley et al. 2001, Zou et al. 2013]
*Permanent analysis*							
rs17226825	9p21.1	33,010,337	− 1.42	7.47e−08	*APTAX* *NFX1*	Non-coding	Repair of DNA damage in cells [Sano et al. 2004] Regulating the duration of an inflammatory response [Yamashita et al. 2016]
rs6708025	2p24.2	178,577,642	− 3.00	1.36e−07	*PDE11A*	Intronic	Messenger in different signal transduction pathways [Fawcett et al. 2000]
rs11686767	2p16.1	60,990,128	− 0.63	3.09e−07	*PAPOLG* *REL*	Intronic	Catalyzing template-independent extension of a DNA/RNA strand [Topalian et al. 2001] Regulate inflammation, immune response & apoptosis [Shono et al. 2014]
rs288547958	7q35	145,203,344	− 176	6.93e−07	*CNTNAP2*	Non-coding	Role in neuronal migration, dendritic arborisation and synaptic transmission [Anderson et al. 2012]
rs73753796	6q15	92,190,076	− 2.91	1.079e−06	*MIR4643*	Non-coding	Involved in post-transcriptional regulation of gene expression [Ghanbari et al. 2015]
rs111979811	7q34	141,658,200	− 2.07	1.11e−06	*TAS2R38,* *TAS2R3,* *TAS2R4,* *TASR25* *OR9A4*	Non-coding	Linked to loci influence bitter perception. Significant association for caries risk [Wendell et al. 2010] Activate the neural response that initiate the perception of smell [Niimura, 2012]

^*^Although no known role of some of the presented genes in cariogenesis and/or odontogenesis, further investigations is needed to discover if some of these genes could have possible role in dental caries and/or odontogenesis

**Fig. 2:**
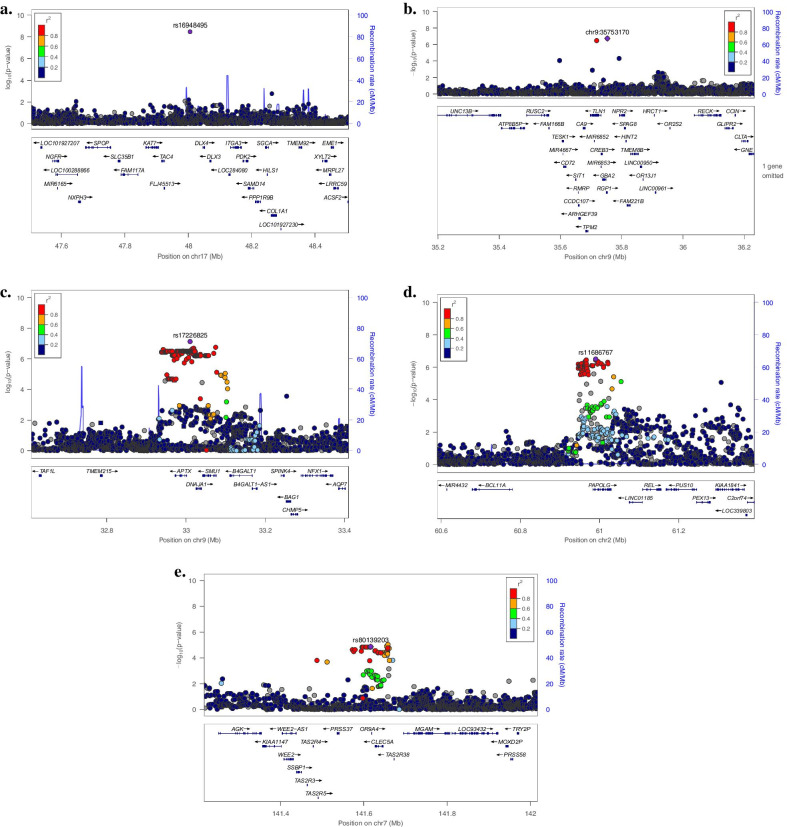
LocusZoom of regions of Interest. Panels a, b and c are association results from the primary dentition GWAS, panels d and e are from the permanent dentition GWAS. **a** suggestive locus near *DLX3* and *DLX4* genes on chromosome 17. **b** suggestive locus near *TLN1* and *CA9* on chromosome 9. **c** suggestive locus near *NFX1* gene on chromosome 9. **d** suggestive locus near *REL* gene on chromosome 2. **e** suggestive locus near Taste receptor genes (*TAS2R38, TAS2R3, TAS2R4, TASR25*) on chromosome 7. The genome build used for the recombination rate was based on 1000 Genomes November 2014 EUR data. All of the gene positions and directions of transcription are annotated on the plots

One of the strongest suggestive association signals for the primary *dft* was at 17q21.33 (rs16948495, *P* = 5.16 × 10^−8^) which is less than 100 kb downstream from *DLX3* and upstream from *DLX4* (see Fig. 2a). These genes belong to the Distal-less homeobox family, known to be expressed in cranial neural crest cells and in the craniofacial mesenchyme; and important in tooth development [18–20]. *DLX3*, which plays an essential role in skeletal formation and development, is also required for tooth morphogenesis [21, 22]. Furthermore, a recent study also showed that *DLX3* plays an important role in dentinogenesis. Mutations in this gene cause tricho-dento-osseous (TDO) syndrome, a rare syndrome that affects the teeth and bones, including enamel hypoplasia and dentin hypoplasia [23]. These severe outcomes of *DLX3* mutations emphasize the important role *DLX3* plays in the development of bones and teeth.

*DLX4* is highly expressed in human dental pulp cells (DPCs) and is associated with abnormal human tooth formation [24]. *DLX4* also plays an important role in forebrain and craniofacial and development. A mutation in *DLX4* was reported to cause a non-syndromic form of cleft lip and palate [25]. Rs16948495 shows enhancer chromatin marks in osteoblasts. These lines of evidence suggest that both *DLX3* and *DLX4* may play important roles in the dental caries process by the regulation tooth development [21–23, 25].

There was also a strong suggestive association signal for primary *dft* and a SNP at 9p13.3 (chr9:35,753,170, *P* = 1.89 × 10^−7^, Fig. 2b). The gene *CA9* (*CA IX*) is located less than 200 kb upstream from the lead SNP at this locus: *CA9* encodes a family of enzymes with at least 14 different isoforms that participate in several biological processes including the secretion of saliva and pH homeostasis [26]. In candidate gene studies, a different member of the CA family (*CA VI*) was investigated due to its role in saliva secretion and regulating salivary pH and was found to be significantly associated with dental caries in children [27–29]. When we looked-up *CA6* gene in our association results, we did find a SNP (rs58579969), 300 kb downstream from it. However, this SNP only showed nominal evidence of an association with primary *dft* (*P* = 1.2 × 10^−3^).

Another gene at the 9p13.3 locus is *TLN1;* a cytoskeletal protein that has been implicated in several biological functions, including migration of fibroblasts and osteoclasts [30, 31]. The expression of *tln1* in zebrafish was observed in the craniofacial cartilage structures, including the palate. *Tln1* mutant zebrafish had malformation in their palate and craniofacial muscles, which indicate the possible role of *tln1* in craniofacial morphogenesis [32]. Neither *TLN1* nor *CA9* genes have been implicated in dental caries before, however, their plausible roles in craniofacial morphogenesis and pH regulation could suggest that they may influence cariogenesis.

#### Permanent dentition

In the permanent dentition analysis, there were no SNPs meeting the genome-wide significance level, but there were results with multiple loci that were within the range for the genome-wide suggestive level (5 × 10^−8^ < *P* < 1 × 10^−6^). Summaries of the lead SNPs and nearby genes from the permanent analysis are presented in Table 5.

One of the strongest suggestive association signals for the permanent DFT was at the 9p21.1 locus (rs17226825, *P* = 7.47 × 10^−8^) which is located within *APTAX* (Fig. 2c), and the encoded protein of this gene is involved in the repair of DNA damage in cells [33]; however, no role is currently known for this gene in odontogenesis or dental caries. The lead SNP is a strong eQTL (expression quantitative trait locus) for *APTAX* (per an (eQTL) database [34]. It is also a strong eQTL for other genes (*B4GALT1, SMU1, RP11-54K16.2)* [16]*.* The lead SNP is also approximately 300 kb upstream from the *NFX1* gene, which plays an important role in regulating the duration of inflammatory responses [35].

Another suggestive association signal at 2p16.1 (rs11686767, *P* = 3.11 × 10^−8^), is intronic to *PAPOLG* (Fig. 2d) which plays a role in catalyzing template-independent extension of a DNA/RNA strand [36] The SNP is a strong eQTL for *PAPOLG* [16]*,* however, no known role yet of this gene in odontogenesis or dental caries. Finally, rs11686767 was also 100 kb upstream to *REL* gene. The protein encoded by this gene, proto-oncogene c-Rel, is involved in many important cellular processes such as apoptosis, inflammation, and the immune response [37]. Those genes; *NFX1* and *REL* could contribute to dental caries by influencing host susceptibility to oral microorganism.

An interesting suggestive association for the permanent dentition DFT was at 7q34 (lead SNP rs11197981; *P* = 1.11 × 10^−6^), approximately 200 kb downstream of taste receptor genes (*TAS2R38, TAS2R3, TAS2R4, TASR25*) (Fig. 2e), which have plausible roles in dental caries. Specifically, *TAS2R38* as has been associated with dental caries in previous studies [38–41]. Further, taste receptor genes, specifically *TAS2R38*, influence bitter perception and dietary habits that are hypothesized to lead to consuming more sweets in the diet and thus leading to dental caries. The lead SNP at this locus is also within the *OR9A4* gene, which is responsible for activating the neural response that initiates the perception of smell [42].

To further investigate these significant or suggestive SNPs, we used the FUMA platform (Functional Mapping and Annotation of Genome-Wide Association Studies) [43] to prioritize, annotate, and interpret the genomic variants and genes from the GWAS results of both primary and permanent analyses. Gene-based testing using GWAS results was computed by MAGMA (Multi-marker Analysis of GenoMic Annotation) using the default settings implemented in FUMA. After mapping the top SNPs from the GWAS analyses to 19,182 protein coding genes, the genome-wide significance level was calculated based on the number of tested genes and it was set at 0.05/19182 = 2.61 × 10^−6^. However, none of the genes in both analyses reached the genome wide significance level. FUMA results of the risk genomic loci for both analyses are presented in the Additional file 1: Tables S1 and S2.

## Discussion

Dental caries is one of the most common chronic diseases that affects both children and adults, and is influenced by a complex interplay of diet, bacteria, salivary flow, genetic factors and other environmental factors. Untreated dental caries could lead to tooth loss which can negatively impact the oral health quality of life (OHRQoL) because it affects function, aesthetics and satisfaction. OHRQoL is a multidimensional concept that includes the impact of dental health on the individual’s functional well-being, emotional health, satisfaction, and self-esteem. OHRQoL is very important because of its usefulness in addressing the impact of oral health disparities and access to care on overall health and quality of life to communicate it with policymakers to improve oral health by increasing access to care [44, 45].

There are now a number of effective preventive methods for dental caries (good oral hygiene, balanced diet, regular dental care) [46, 47]. Most notably, stannous fluoride, such as in toothpaste and water, has been an important public health advance by reducing the prevalence of dental caries [48]. However, dental caries is still wide spread worldwide [3], requiring additional approaches to understanding risk factors for dental caries.

The current study took a genome-wide approach to investigate genetic factors contributing to dental caries in the primary and permanent dentitions. Several previous genetic studies have investigated and identified the role of different genes in cariogenesis. Most of these past studies involved samples that were ethnically homogeneous, mainly of European descent [49–53], unlike the current multiethnic study (see also Table 1).

Notably, results in the primary dentition nominated a few SNPs that showed association at genome-wide significance, and multiple loci that showed suggestive evidence for association. In the permanent dentition, no variants reached genome-wide significance, but several reached suggestive significances. Importantly, none of the top associated SNPs in either of the analyses (*dft* and DFT) were replicated in the other one, full results are presented in the Additional file 1: Tables S3 and S4. This confirms that genetic loci that influence the susceptibility to dental caries differ between primary and permanent dentitions [54]. Although none of the genome-wide significant association signals were near genes with known roles in dental caries or tooth development, some of the suggestive signals were near genes with potential roles in dental caries.

Some of the new associations identified in this study were in or near genes that have important roles in the inflammatory process, such as *NFX1* and *REL* genes (permanent dentition). Those genes could contribute to dental caries by influencing host susceptibility to oral microorganism. This study also identified association signals near genes that have a confirmed role in tooth and craniofacial development, such as *TLN1* and *DLX* genes. Heterozygous variants in *DLX4* have been implicated before in Orofacial clefting [25]. This could indicate that both dental caries and OFC share common genetic risk loci. This is plausible because some of the genes that had been previously implicated in dental caries and OFCs are craniofacial development genes. *PKD2* gene is one of the genes that has been associated with dental caries and had an implication in craniofacial development [8] and mutation in *PKD2* has been linked to craniofacial anomalies and tooth loose in mice [55]. Thus, our future investigations will focus on exploring the relationship between dental caries and OFCs on both phenotypic and genetic levels.

This study also supported the role of taste receptor genes in dental caries (*TAS2R38, TAS2R3, TAS2R4, TASR25,* see Table 5), which have been associated with dental caries in previous studies [38–41]. On the other hand, several loci/genes associated with dental caries from previous large meta-GWAS studies of dental caries [50, 53] were reviewed in our results (the lead SNPs ± 500 kb, a total of 5935 SNPs), but none of these variants reached statistical significance (see Additional file 1: Tables S5, S6 for details).

In the permanent dentition subset of the current study population, females had a significantly higher rate of dental caries than males (*P* = 0.000234) consistent with earlier studies [56, 57]. However, there was no gender difference in the primary dentition. Possible explanations for the increased rates of dental caries among adult females include (i) the fact that teeth tend to erupt earlier in females, which allows for a longer exposure to a cariogenic oral environment, and (ii) hormonal changes during pregnancy affect saliva pH leading to a decrease in the concentration of calcium, phosphorus, magnesium, and chloride, which, in turn might lead to increased caries. (iii) other behavioral/environmental factors during pregnancy such as consuming a diet high in sucrose and poor oral hygiene could also increase the risk of developing more dental caries.

## Conclusion

In summary, this study found multiple associations near genes that may play important roles in dental caries, such as *TAS2R38, TAS2R3, TAS2R4, TASR25*, *DLX3* and *DLX4,* several of which have plausible biological functions relevant to tooth development and cariogenesis. These findings contribute to our understanding of the genetic mechanisms of cariogenesis, providing targets for follow-up translational studies that will eventually improve prevention and treatment of this highly prevalent chronic disease worldwide.

## Supplementary Information

**Additional file 1**. Supplementary material.

## Data Availability

The datasets generated and/or analysed during the current study are available in the dbGaP repository, https://www.ncbi.nlm.nih.gov/projects/gap/cgi-bin/study.cgi?study_id=phs000774.v2.p1

## Abbreviations

dft: Number of decayed or filled primary tooth
DFT: Number of decayed or filled permanent teeth
GWAS: Genome-wide association study
MAF: Minor allele frequency
SNP: Single nucleotide polymorphism
