# Supplementary material for: Genome-Wide Association Study (GWAS) of dental caries in diverse populations
Source: BMC Oral Health. 2021 Jul 26;21:377. doi: 10.1186/s12903-021-01670-5 (PMC8311973; doi:10.1186/s12903-021-01670-5)
Supplement: Supplementary file 1 — Additional file 1. Supplementary material. [file 12903_2021_1670_MOESM1_ESM.docx]

**Genome-Wide Association Study (GWAS) of Dental Caries in Diverse Populations**

Rasha N. Alotaibi^1,2^, Brian J. Howe^3,4^, Jonathan M. Chernus^1,5^, Nandita Mukhopadhyay^1,2^, Carla Sanchez^1,2^, Frederic W.B. Deleyiannis ^6^, Katherine Neiswanger^1,2^, Carmencita Padilla^7^, Fernando A. Poletta^8^, Ieda M. Orioli^9^, Carmen J. Buxó^10^, Jacqueline T. Hecht^11^, George L. Wehby^12^, Ross E. Long^13^, Alexandre R. Vieira^1,^ Seth M. Weinberg^1,2^, John R. Shaffer ^1,2,5^, Lina Moreno Uribe ^3,14^ Mary L. Marazita ^1,2,5^

^1^ Department of Oral and Craniofacial Sciences, School of Dental Medicine, University of Pittsburgh, Pittsburgh, PA, United States

^2^ Center for Craniofacial and Dental Genetics, School of Dental Medicine, University of Pittsburgh, Pittsburgh, PA, United States

^3^ Department of Family Dentistry, College of Dentistry, University of Iowa, Iowa City, IA, United States

^4^ The Iowa Center for Oral Health Research, College of Dentistry, University of Iowa, Iowa City, IA, United States

^5^ Department of Human Genetics, Graduate School of Public Health, University of Pittsburgh, Pittsburgh, PA, United States

^6^UC Health Medical Group, Colorado Springs, Denver, CO, United States

^7^ Department of Pediatrics, College of Medicine, University of the Philippines, Manila

^8^ ECLAMC / INAGEMP at Center for Medical Education and Clinical Research (CEMIC-CONICET), Buenos Aires, Argentina.

^9^ Department of Genetics, Institute of Biology, Federal University of Rio de Janeiro, Rio de Janeiro, Brazil

^10^ Dental and Craniofacial Genomics Core, School of Dental Medicine, University of Puerto Rico, San Juan, Puerto Rico

^11^ Department of Pediatrics, University of Texas Health Science Center at Houston, Houston, TX, United States

^12^ Department of Health Management and Policy, College of Public Health, University of Iowa, Iowa City, IA, United States

^13^ Lancaster Cleft Palate Clinic, Lancaster, PA, United States

^14^ Department of Orthodontics, School of Dentistry, University of Iowa, Iowa City, IA, United States

**Corresponding Author:**

Rasha N. Alotaibi

School of Dental Medicine
Center for Craniofacial and Dental Genetics
University of Pittsburgh
Suite 500 Bridegeside Point​
100 Technology Dr.
Pittsburgh, PA 15219

Tel: +1(412) -802-5587

E-mail: rna11@pitt.edu

**Genome-Wide Association Study (GWAS) of Dental Caries in Diverse Populations**

**Supplementary material**

| **Table S1. FUMA Risk Genomic loci based on the Primary *dft* GWAS analysis results** | | | | | | | | | | | | |
| --- | --- | --- | --- | --- | --- | --- | --- | --- | --- | --- | --- | --- |
| **rsID** | **chr** | **pos** | **A1** | **A2** | **MAF** | **gwasP** | **nearestGene** | **Func^a^** | **CADD^b^** | **RDB^c^** | **Min**  **ChrState^d^** | **Common**  **ChrState^e^** |
| rs80177293 | 5 | 88865345 | C | T | 0.05431 | 7.86E-10 | MIR3660 | intergenic | 4.061 | 5 | 5 | 15 |
| rs113021760 | 9 | 114805385 | T | G | 0.05351 | 5.49E-09 | SUSD1 | intronic | 2.641 | 6 | 4 | 5 |
| rs2296213 | 9 | 114820741 | A | G | 0.05252 | 1.39E-07 | SUSD1 | exonic | 10.84 | NA | 4 | 5 |
| rs117298946 | 9 | 114821364 | A | G | 0.05292 | 2.14E-07 | SUSD1 | intronic | 0.236 | 7 | 4 | 5 |
| ^a^func : Functional consequence of the SNP on the gene obtained from ANNOVAR.  ^b^CADD: CADD score which is computed based on 63 annotations. The higher the score, the more deleterious the SNP is.  ^c^RDB: RegulomeDB score which is a categorical score (from 1a to 7).  ^d^minChrState: The minimum 15-core chromatin state across 127 tissue/cell type.  ^e^commonChrState: The most common 15-core chromatin state across 127 tissue/cell types. | | | | | | | | | | | | |

| **Table S2. FUMA Risk Genomic loci based on the Permanent DFT GWAS analysis results** | | | | | | | | | | | | |
| --- | --- | --- | --- | --- | --- | --- | --- | --- | --- | --- | --- | --- |
| **rsID** | **chr** | **pos** | **A1** | **A2** | **MAF** | **gwasP** | **nearestGene** | **Func^a^** | **CADD^b^** | **RDB^c^** | **minChr**  **State^d^** | **Common**  **ChrState^e^** |
| rs17226825 | 9 | 33010337 | T | C | 0.03474 | 7.47E-08 | APTX | intronic | 8.545 | 5 | 4 | 15 |
| rs6708025 | 2 | 178577642 | A | C | 0.1 | 1.36E-07 | PDE11A | ncRNA_intronic | 2.856 | 6 | 5 | 15 |
| ^a^func : Functional consequence of the SNP on the gene obtained from ANNOVAR.  ^b^CADD: CADD score which is computed based on 63 annotations. The higher the score, the more deleterious the SNP is.  ^c^RDB: RegulomeDB score which is a categorical score (from 1a to 7).  ^d^minChrState: The minimum 15-core chromatin state across 127 tissue/cell type.  ^e^commonChrState: The most common 15-core chromatin state across 127 tissue/cell types. | | | | | | | | | | | | |

| **Table S3. Lead SNPs from the Primary *dft* GWAS in the Permanent DFT GWAS results** | | | | | | | | | | | |
| --- | --- | --- | --- | --- | --- | --- | --- | --- | --- | --- | --- |
| **SNP** | **CHR** | **BP** | **A1** | **A2** | **P_Prim** | **BETA_Prim** | **MAF_Prim** | **P_Perm** | **BETA_Perm** | **MAF_Perm** | **Type** |
| rs80177293 | 5 | 88865345 | C | T | 7.86E-10 | -2.9159954 | 0.02118 | 0.55101424 | -0.21848 | 0.02118 | Imputed |
| rs113021760 | 9 | 114805385 | T | G | 5.49E-09 | -2.6746476 | 0.02389 | 0.90106888 | 0.04709507 | 0.02389 | Imputed |
| rs75833698 | 6 | 19866520 | T | C | 5.01E-08 | -2.169268 | 0.02958 | 0.91083619 | -0.0339142 | 0.02958 | Imputed |
| rs16948495 | 17 | 48005748 | A | G | 5.24E-08 | -2.1518084 | 0.03894 | 0.64906346 | -0.1484511 | 0.03894 | Imputed |
| rs75459295 | 14 | 34767100 | G | A | 5.99E-08 | -2.2560519 | 0.03228 | 0.88718769 | -0.0470517 | 0.03228 | Imputed |
| rs202021750 | 13 | 44515845 | C | CTCTATCTA | 1.58E-07 | -1.8260505 | 0.03276 | 0.78042293 | 0.07769769 | 0.03276 | Imputed |
| chr9:35753170 | 9 | 35753170 | A | C | 1.89E-07 | -2.2735767 | 0.02823 | 0.29097557 | 0.34571346 | 0.02823 | Genotyped |
| rs78046168 | 4 | 141998818 | G | A | 3.17E-07 | -2.311912 | 0.02668 | 0.82188451 | 0.08220072 | 0.02668 | Imputed |
| rs11030361 | 11 | 28543199 | G | A | 3.48E-07 | -2.0906908 | 0.04222 | 0.08698979 | -0.5272759 | 0.04222 | Imputed |
| rs199793306 | 17 | 8023061 | T | C | 3.59E-07 | -1.8783185 | 0.03284 | 0.66343266 | 0.14191645 | 0.03284 | Imputed |

| **Table S4. Lead SNPs from the Permanent DFT GWAS in the Primary *dft* GWAS results** | | | | | | | | | | | |
| --- | --- | --- | --- | --- | --- | --- | --- | --- | --- | --- | --- |
| **SNP** | **CHR** | **BP** | **A1** | **A2** | **P_Perm** | **BETA_Perm** | **MAF_Perm** | **P_Prim** | **BETA_Prim** | **MAF_Prim** | **Type** |
| rs17226825 | 9 | 33010337 | T | C | 7.47E-08 | -1.4179267 | 0.05272 | 0.66358054 | 0.12526288 | 0.05272 | Imputed |
| rs6708025 | 2 | 178577642 | A | C | 1.36E-07 | -3.0037507 | 0.03345 | 0.11079061 | -0.9272083 | 0.03345 | Imputed |
| rs11686767 | 2 | 60990128 | G | A | 3.10E-07 | -0.6312099 | 0.4088 | 0.45578658 | -0.1047657 | 0.4088 | Imputed |
| rs28854795 | 7 | 145203344 | T | C | 6.93E-07 | -1.7629992 | 0.04691 | 0.41571172 | -0.3360052 | 0.04691 | Imputed |
| rs73753796 | 6 | 92190076 | C | T | 1.08E-06 | -2.9121337 | 0.02094 | 0.86399582 | 0.08795262 | 0.02094 | Imputed |
| rs111979811 | 7 | 141658200 | G | A | 1.11E-06 | -2.0752691 | 0.02959 | 0.93936798 | 0.03435144 | 0.02959 | Imputed |
| rs4305230 | 2 | 29812191 | T | C | 1.12E-06 | -0.5759623 | 0.4174 | 0.54478259 | -0.0800947 | 0.4174 | Imputed |
| rs13333858 | 16 | 52490111 | G | A | 1.45E-06 | -1.9914971 | 0.02808 | 0.81770171 | -0.0952939 | 0.02808 | Imputed |
| rs114530407 | 3 | 75525724 | G | A | 1.91E-06 | -1.3337292 | 0.0507 | 0.84186328 | -0.063533 | 0.0507 | Imputed |
| rs11018162 | 10 | 129306652 | C | T | 1.99E-06 | -1.77809 | 0.02384 | 0.08472631 | 0.80020232 | 0.02384 | Imputed |

| **Table S5. Association Results for The Previously Identified Variants Near Dental Caries Genes in Prim *dft* analysis** | | | | | | | | | |
| --- | --- | --- | --- | --- | --- | --- | --- | --- | --- |
| **Gene** | **Previous SNP** | **Best SNP at this locus^a^** | **CHR** | **BP** | **P^b^** | **BETA** | **A1** | **A2** | **MAF** |
| *KRTCAP2* | rs4971099 | rs12126509 | 1 | 155155608 | 0.08 | -0.08 | A | G | 0.45 |
| *WNT10A* | rs121908120^c^ | rs730947 | 2 | 219703298 | 0.0003 | 0.97 | C | A | 0.089 |
| *C5orf66* | rs1122171 | rs1122171 | 5 | 134509987 | 0.02 | 0.29 | C | T | 0.43 |
| *FGF10* | rs1482698 | rs12187122 | 5 | 44539453 | 0.09 | -0.01 | C | G | 0.36 |
| *HLA* | rs9366651 | chr6:26383042 | 6 | 26336696 | 0.02 | -0.09 | G | T | 0.41 |
| *PBX3* | rs10987008 | rs1411352 | 9 | 128661600 | 0.13 | 0.04 | T | A | 0.40 |
| *CA12* | rs72748935 | rs34588257 | 15 | 63639416 | 0.003 | 0.34 | C | T | 0.35 |
| *FOXL1* | rs10048146 | rs12929537 | 16 | 86710660 | 0.001 | 0.37 | G | A | 0.15 |
| *MC4R* | rs28822480 | rs9948863 | 18 | 57924823 | 0.02 | 0.02 | A | G | 0.19 |
| *MAMSTR* | rs11672900 | rs653395 | 19 | 49220323 | 0.0003 | -0.26 | G | A | 0.31 |
| *ALLC* | rs1594318 | rs201754110 | 2 | 3733944 | 0.02 | -0.27 | G | C | 0.33 |
| *ALLC* | rs872877 | rs35498072 | 2 | 3735826 | 0.04 | -0.31 | G | A | 0.34 |
| *NEED9* | rs7738851 | rs12470798 | 6 | 11241788 | 0.002 | 0.16 | T | A | 0.23 |
| *Variants shown in this table is from previous GWAS meta-analysis of dental caries in different populations [Haworth et al., 2018, Shungin et al., 2019]  ^a^ Best SNP defined by +/- 500kb region around previously reported SNP  ^b^ Bonferroni corrected alpha for significance = 0.05/5935= 8.4246e-06  ^c^ No association results were found for this variant in our results | | | | | | | | | |

| **Table S6. Association Results for The Previously Identified Variants Near Dental Caries Genes in Perm DFT analysis** | | | | | | | | | | |
| --- | --- | --- | --- | --- | --- | --- | --- | --- | --- | --- |
| **Gene** | **Previous SNP** | **Best SNP at this locus^a^** | **CHR** | **BP** | **P^b^** | **BETA** | **A1** | **A2** | **MAF** | |
| *KRTCAP2* | rs4971099 | rs72204645 | 1 | 155155608 | 0.0003 | -0.17 | TAC | T | 0.4587 | |
| *WNT10A* | rs121908120^c^ | rs11902348 | 2 | 219708638 | 0.001 | -1.92 | G | A | 0.0255 | |
| *C5orf66* | rs1122171 | rs4246777 | 5 | 134519009 | 0.002 | 0.25 | G | T | 0.439 | |
| *FGF10* | rs1482698 | rs72750292 | 5 | 44574965 | 0.02 | -0.19 | A | G | 0.3646 | |
| *HLA* | rs9366651 | rs11643948 | 6 | 86740411 | 0.005 | -0.18 | C | T | 0.4137 | |
| *PBX3* | rs10987008 | rs201592676 | 9 | 128635466 | 0.06 | 0.04 | AAAAAC | A | 0.4096 | |
| *CA12* | rs72748935 | rs200117093 | 15 | 63591643 | 0.009 | -0.26 | AC | A | 0.3525 | |
| *FOXL1* | rs10048146 | rs11643948 | 16 | 86740411 | 0.009 | 0.19 | C | T | 0.1539 | |
| *MC4R* | rs28822480 | exm-rs489693^d^ | 18 | 57882787 | 0.04 | -0.19 | A | G | 0.1965 | |
| *MAMSTR* | rs11672900 | rs28746180 | 19 | 49202449 | 0.0003 | -0.31 | T | TTTT | 0.3154 | |
| *ALLC* | rs1594318 | rs370890406 | 2 | 3719602 | 0.02 | 0.095 | G | T | 0.33 | |
| *ALLC* | rs872877 | rs7576701 | 2 | 3735826 | 0.03 | 0.13 | G | A | 0.34 | |
| *NEED9* | rs7738851 | rs72783478 | 6 | 11213997 | 0.009 | 0.028 | C | T | 0.23 | |
| *Variants shown in this table is from previous GWAS meta-analysis of dental caries in different populations [Haworth et al., 2018, Shungin et al., 2019]  ^a^ Best SNP defined by +/- 500kb region around previously reported SNP  ^b^ Bonferroni corrected alpha for significance = 0.05/5935= 8.4246e-06  ^c^ No association results were found for this variant in our results  ^d^ This is the only genotyped SNP in the table | | | | | | | | | |  |
